# Local Public Health System Response to the Tsunami Threat in Coastal California following the Tōhoku Earthquake

**DOI:** 10.1371/4f7f57285b804

**Published:** 2012-07-16

**Authors:** Jennifer C. Hunter, Adam W. Crawley, Michael Petrie, Jane E. Yang, Tomás J. Aragón

## Abstract

Background On Friday March 11, 2011 a 9.0 magnitude earthquake triggered a tsunami off the eastern coast of Japan, resulting in thousands of lives lost and billions of dollars in damage around the Pacific Rim. The tsunami first reached the California coast on Friday, March 11th, causing more than $70 million in damage and at least one death. While the tsunami’s impact on California pales in comparison to the destruction caused in Japan and other areas of the Pacific, the event tested emergency responders’ ability to rapidly communicate and coordinate a response to a potential threat. Methods To evaluate the local public health system emergency response to the tsunami threat in California, we surveyed all local public health, emergency medical services (EMS), and emergency management agencies in coastal or floodplain counties about several domains related to the tsunami threat in California, including: (1) the extent to which their community was affected by the tsunami, (2) when and how they received notification of the event, (3) which public health response activities were carried out to address the tsunami threat in their community, and (4) which organizations contributed to the response. Public health activities were characterized using the Centers for Disease Control and Prevention (CDC) Public Health Preparedness Capabilities (PHEP) framework. Findings The tsunami's impact on coastal communities in California ranged widely, both in terms of the economic consequences and the response activities. Based on estimates from the National Oceanic and Atmospheric Administration (NOAA), ten jurisdictions in California reported tsunami-related damage, which ranged from $15,000 to $35 million. Respondents first became aware of the tsunami threat in California between the hours of 10:00pm Pacific Standard Time (PST) on Thursday March 10th and 2:00pm PST on Friday March 11th, a range of 16 hours, with notification occurring through both formal and informal channels. In response to this threat, the activities most commonly reported by the local government agencies included in this study were: emergency public information and warning, emergency operations coordination, and inter-organizational information sharing, which were reported by 86%, 75%, and 65% of all respondents, respectively. When looking at the distribution of responsibility, emergency management agencies were the most likely to report assuming a lead role in these common activities as well as those related to evacuation and community recovery. While activated less frequently, public health agencies carried out emergency response functions related to surveillance and epidemiology, environmental health, and mental health/psychological support. Both local public health and EMS agencies took part in mass care and medical material management activities. A large network of organizations contributed to response activities, with emergency management, law enforcement, fire, public health, public works, EMS, and media cited by more than half of respondents. Conclusions In response to the tsunami threat in California, we found that emergency management agencies assumed a lead role in the local response efforts. While public health and medical agencies played a supporting role in the response, they uniquely contributed to a number of specific activities. If the response to the recent tsunami is any indication, these support activities can be anticipated in planning for future events with similar characteristics to the tsunami threat. Additionally, we found that many respondents first learned of the tsunami through the media, rather than through rapid notification systems, which suggests that government agencies must continue to develop and maintain the ability to rapidly aggregate and analyze information in order to provide accurate assessments and guidance to a potentially well-informed public. Citation: Hunter JC, Crawley AW, Petrie M, Yang JE, Aragón TJ. Local Public Health System Response to the Tsunami Threat in Coastal California following the Tōhoku Earthquake. PLoS Currents Disasters. 2012 Jul 16

## Background

On Friday March 11, 2011 a 9.0 magnitude earthquake triggered a tsunami off the eastern coast of Japan, resulting in thousands of lives lost and billions of dollars in damage around the Pacific Rim. The tsunami first reached the California coast at approximately 7:30am on Friday, March 11th, with a wave height of up to eight feet, causing more than $70 million in damage and at least one death.^1^ While the tsunami’s impact on California pales in comparison to the destruction caused in Japan and other areas of the Pacific, the event tested emergency responders’ ability to rapidly communicate and coordinate a response to a potential threat. The Pacific coast of the United States is recognized as a tsunami-prone region, but actual tsunami events, and opportunities to learn from them, are infrequent. Fewer than twenty other damaging tsunamis have been recorded in this region in the past century, three quarters of which occurred in the state of Alaska.^2^ To our knowledge, none of these events have been studied in terms of the overall public health response. Therefore, the recent tsunami event presents a unique opportunity for researchers to describe local public health systems’ response using a systematic retrospective analysis. The use of such facilitated look-backs has been recognized as a potential method of preparedness assessments and improvements.^3^ ^4^ ^5^

**Tsunami Travel Times:**
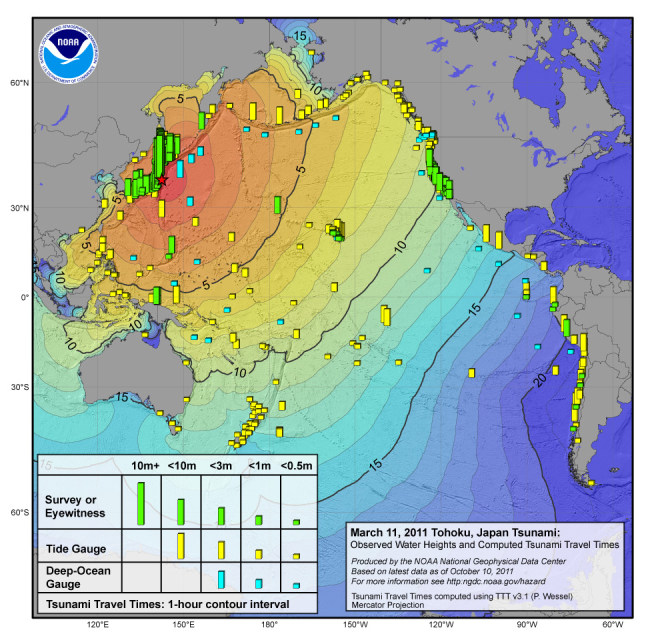
Source: NOAA/National Geophysical Data Center^1^

This study describes the key public health emergency functions activated by local public health and emergency medical governmental agencies in response to the tsunami threat to the California coast. We also characterize local emergency management activities to put public health activities in the context of the overall response. Additionally, we describe how and when affected agencies became aware of the tsunami threat in California and identify challenges and lessons learned from the response.

## Methods

**Study population.** To evaluate the local public health emergency response to the tsunami threat in California, our target study population included all local public health, emergency medical services, and emergency management agencies in coastal or floodplain counties. By surveying representatives from public health and emergency medical services agencies, we attempted to capture the experiences of the local governmental agencies responsible for medical and public health response. Local emergency management agencies were included for the purpose of contextualizing the governmental medical and health activities within the overall response. Therefore, the target population consisted of all local health departments (LHDs; n=21), all local emergency medical services (EMS) agencies (n=18), and all local emergency management agencies or offices of emergency services (OES; n=18) in coastal and floodplain counties. Two of these counties are home to California’s three city health departments, which accounts for the higher number of LHDs surveyed. Representatives from each organization were chosen based on their functional role: health officials for LHDs, administrators for local EMS agencies, and directors for OES agencies.

**Measures and Data Collection Instrument.** Using a web-based survey we asked respondents about several domains related to the tsunami threat in California, including: (1) the extent to which their community was affected by the tsunami (e.g., damage, injuries, and deaths), (2) when and how they received notification of the event, (3) which public health response activities were carried out to address the tsunami threat in their community, and (4) which organizations contributed to response activities. Additionally, participants were asked about what went well, challenges faced, and lessons learned during the response. Participants were not asked about public concern over potential radiation exposure as a result of the heavily damaged Fukushima Daiichi Nuclear Power Plant in Japan (see Appendix 1 for link to survey instrument).

We measured public health response activities using the CDC Public Health Preparedness Capabilities framework.^6^ This instrument, developed through a review of legislative and executive directives, expert panel processes, and extensive stakeholder engagement, identifies and defines 15 types of services that public health systems could be expected to deliver during emergencies. Four additional sets of response activities were included in the assessment based on researchers’ interests and functions expected during a tsunami threat, including: evacuation, environmental health investigation, mental health/psychological support, and veterinary/animal health investigation or support. Participants were asked to self-report whether each activity was performed in their jurisdiction. For each of the activated functions, participants were asked to rate the extent to which their agency was responsible for carrying out the activity using a 5-point Likert scale ranging from “not at all” to “completely”.

As a proxy measure of agency notification time, participants were asked to self-report when they first became aware of the tsunami threat in California and when they formally received notification of the threat based on their role in their organization or agency. Participants were also asked from what source and medium they first became aware of the threat and which sources their agency used to maintain situational awareness throughout the response.

The survey was reviewed by a practice-based Research Steering Committee, a group composed of state and local agency representatives from public health, emergency medical services, and hospitals. Additional modifications were made based on this feedback.

Organizational representatives were invited via email to participate in the web-based survey, which was created and administered via Qualtrics^©^. All survey recipients were provided a short description of the survey goals from the office of the Principal Investigator of Cal PREPARE, a CDC Preparedness and Emergency Response Research Center at the University of California, Berkeley. Survey recipients were asked to complete the survey or to forward the survey link to the person most knowledgeable of their agency’s response; thus, only one survey response should have been received from each agency. Survey data collection took place over the course of four weeks, beginning on August 25, 2011 and ending on November 10, 2011. Two email reminders were sent to invited participants in effort to promote higher response rates.

**Data analysis.** Survey data were downloaded from the web survey provider and analyzed using both Qualtrics and Stata 11 (StataCorp LP, College Station, TX). A descriptive analysis of quantitative survey data is presented here with figures to illustrate the public health capability profiles. Qualitative data from the survey, including open-ended questions regarding lessons learned from the event, were independently coded by two researchers (AC, JY) to classify the statements into categories and themes. Codes were compared and all discrepancies were resolved. A summary of themes is presented here with quotations from open-ended survey questions to illustrate salient issues.*

This research was approved by the Committee for Protection of Human Subjects at the University of California, Berkeley.

## Results

**Survey Response Rate and Respondent Demographics.** Survey responses were received from thirty-two agencies in total, including twelve LHDs, eleven local EMS agencies, and nine OES agencies, representing response rates of 57%, 61%, and 50%, respectively, of the recruited agencies. Seventeen cities and counties were represented out of a possible twenty-one, representing a response rate of 81% by jurisdiction. Chi-square tests indicated that responding counties did not differ significantly (at the p<0.05 threshold) from non-responding counties in terms of county size and geography, with county size defined as a categorical variable of small (serving fewer than 50,000 people), medium (serving between 50,000 and 500,000 people) and large (serving more than 500,000 people) and geography defined according to California’s Mutual Aid Regions.^7^ Analysis using a t-test indicated that response rates did not vary significantly (at the p<0.05 level) for median income. The characteristics of responding and non-responding agencies are reported in Table 1.

**Table 1. Characteristics of Responding and Non-Responding Agencies:** 

	Responding Agencies			Non-Responding Agencies		
**Agency Type**	LHDs (n=12)	Local EMS Agencies (n=11)	Local OESs (n=9)	LHDs (n=9)	Local EMS Agencies (n=7)	Local OESs (n=9)
**Median Population Size Served** ^a^	636,783	753,197	458,614	399,347	401,762	753,197
**Average Median Household Income of Jurisdiction** ^a^	$48,098	$50,818	$51,034	$51,163	$50,125	$50,063
**County Size** ^b^						
Small	0%	9%	0%	11%	0%	11%
Medium	42%	27%	56%	56%	72%	33%
Large	58%	64%	44%	33%	28%	56%
**Geography** ^c^						
Coastal	42%	55%	67%	78%	72%	56%
Southern	50%	45%	33%	22%	14%	33%
Inland	8%	0%	0%	0%	14%	11%
^a^ Based on data from the U.S. Census Bureau, 2000. ^b^ Small population <50,000; Medium population 50,000 to 500,000; Large population >500,000 ^c^ Based on California's Mutual Aid Regions						

The most common functional roles of respondents were: agency administrator/director (50% of total), health officer/deputy health officer (32% of total), and emergency preparedness coordinator (21% of total). Two respondents served in more than one role (director/health officer; director/preparedness coordinator). The functional role of survey respondents did not systematically vary by agency type.

**Damage, Injuries, and Deaths Reported.** Seven of nine OES agency respondents reported tsunami-related damage in their operational area, including physical and business damage. Of responding jurisdictions, damage estimates ranged from very limited (less than $100,000) to extreme (more than $25 million), with median damage estimated at approximately $325,000.^1^ The distribution of damage sites across California is shown in Figure 2. No agencies noted damage to the medical and/or health system.

Based on respondents’ accounts, one death and two injuries were associated with the tsunami. One man was swept out to sea while photographing the event near the mouth of the Klamath River (in Northern California). Two men suffered internal injuries when they were thrown from their boat during the tsunami.

**Damage to California coastal areas from the 2011 tsunami event:**
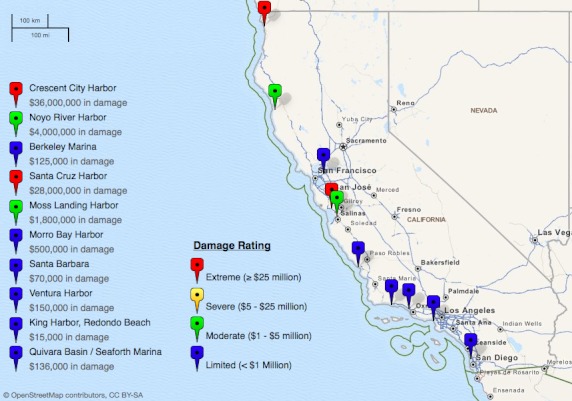
Note that damage points on the above map are not indicative of survey participants. Source: NOAA/National Geophysical Data Center^1^ . This map was developed using GeoCommons data visualization software.

**Notification & Alerting.** Participants were asked a series of questions about when and how they became aware of the tsunami threat in California. Specifically, participants were asked when they first became aware of the threat and when they formally received notification of the threat based on their role in their organization or agency. These notification time results are depicted in Figure 3. In summary, respondents reported first becoming aware of the tsunami threat in California between the hours of 10:00pm on Thursday March 10^th^ and 2:00pm on Friday March 11^th^, a range of 16 hours. The average time when respondents first became aware of the threat was approximately 1:30am on Friday March 11^th^, with the average time of formal agency notification occurring at approximately 2:30am. Public health respondents, on average, learned about the threat later than their EMS and OES counterparts, through both formal and informal channels.^§^ Two respondents (both from LHDs) reported that they did not receive formal notification of the tsunami threat in California, while three public health respondents only received formal notification of the tsunami threat after the event occurred.

**Time of notification of tsunami threat in California, by agency:**
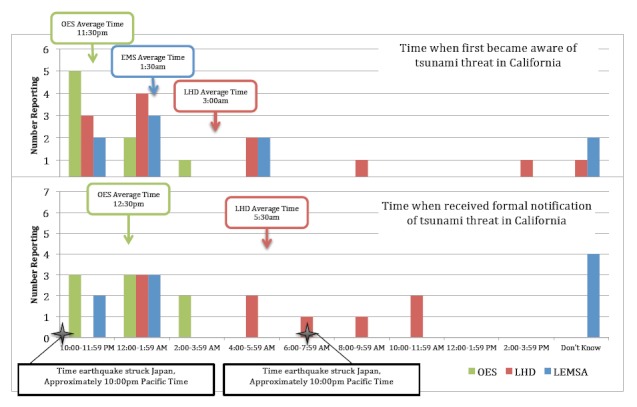

Respondents were also asked from what person or organization they first learned of the tsunami threat in California. The majority of respondents first learned of the threat from either the television news media (29%), from the California Department of Public Health (CDPH) via its messages through the California Health Alert Network (25%), or the California Emergency Management Agency (Cal EMA; 21%). Alerts from CDPH and Cal EMA were received via text messages, email, and telephone. Other respondents reported first learning about the threat from local OESs, the National Oceanic and Atmospheric Administration (NOAA), and other local governmental officials (all cited by three or fewer respondents). While respondents from all agency types reported using the media and Cal EMA as an initial information source, only EMS and public health representatives cited CDPH as their initial source of information.

Eighteen respondents (representing nineteen agencies) indicated that they alerted other organizations or entities about the tsunami threat in California once they became aware of it, including all responding OES agencies and nearly half of responding local EMS agencies and LHDs. Among the agencies that alerted other organizations, local OES agencies were the most active. The nine OES agencies that participated reported notifying other government agencies (100%), the media (55%), non-governmental critical infrastructure (27%), healthcare providers and/or delivery systems (18%), universities (18%), community or faith-based organizations (18%), businesses (18%), and tribal entities (11%). Local EMS agencies and LHDs played a smaller role in notifying other agencies, most often notifying other government agencies as well as healthcare providers and/or delivery systems. Overall, other government agencies and healthcare providers and/or delivery systems were the types of organizations most often notified by responding agencies.

**Situational Awareness.** The most common sources of situational awareness information throughout the tsunami event were Cal EMA or the state warning center (43%), local OES (36%), CDPH (32%), NOAA (29%), and traditional media (25%). Agencies differed in their sources of information, with OES agencies more commonly receiving information from Cal EMA (78% of OES agencies versus 17% of LHDs), LHDs frequently relying on CDPH (50% of LHDs versus 0% of OES), and local EMS agencies often accessing information from various sources including traditional media (67%), local OES (44%), CDPH (44%), and NOAA (33%).

**Response Capabilities Activated.** Using the CDC Public Health Preparedness Capabilities as a framework, respondents were asked what activities were performed in their operational area during the tsunami threat to California, regardless of whether their own agency played a role in that activity. Across all agency types, the most common response functions included emergency public information and warning, emergency operations coordination, and inter-organizational information sharing, which were reported by 86%, 75%, and 65% of all respondents, respectively. Respondents were also asked to describe their agency’s level of involvement in any of the activated response capabilities, shown in Figure 4. Only those activities for which respondents indicated that their agency had some responsibility in performing are shown. In summary, participants from OES agencies were most likely to have reported that their agency had complete (or near complete) responsibility for emergency operations coordination (100%), emergency public information and warning (89%), inter-organizational information sharing (78%) and evacuation activities (56%). Participants from public health agencies uniquely activated emergency response functions such as surveillance and epidemiology, environmental health, and mental health/psychological support. Local EMS agencies uniquely reported participating in mass care activities. Both public health and EMS agencies also contributed to management and distribution of medical materials.

**Activated Public Health Emergency Preparedness functions, by agency type and level of involvement:**
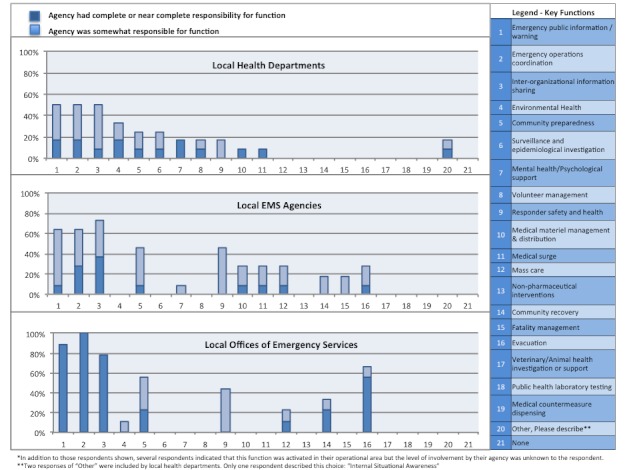

**Response System & Emergency Response Structure.** Participants were asked about the organizations and agencies that contributed to response activities. Government agencies and the media were most commonly identified, cited by 85% and 55% of respondents, respectively. As shown in Figure 5, the government agencies most commonly contributing to response activities involved emergency management, law enforcement, fire, public health, EMS, and public works. For some counties, a wide range of partners contributed to the tsunami response. The average number of partnering agencies was highest for OES agencies (mean: 9.2, range: 3-19), followed by LHDs (mean: 7.6, range: 0-21), and EMS agencies (mean: 7.3, range: 0-15).

**Types of government agencies that contributed to the tsunami response, as reported by survey respondents:**
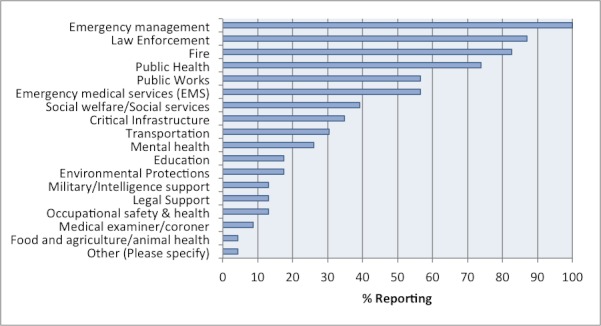

Thirteen operational areas reported activating their County Emergency Operations Center (EOC), with 62% of these operational areas also reporting the activation of one or more City EOCs. Three OES agencies, one local EMS agency, and three LHDs also reported activating their Departmental Operations Centers (DOC); all seven organizations were located in operational areas where the EOC was activated.

**Challenges and Lessons Learned.** Participants were specifically asked whether their agency had experienced any challenges related to information management during the response. Fourteen respondents reported experiencing such challenges, the most common of which were: (1) an insufficient number of trained personnel (36%), (2) the inability to maintain situational awareness (36%), (3) excessive communications workload (36%), and insufficient guidance for inter-organizational information sharing (29%).^?^ Additional reported challenges included communications equipment or system failures, inadequate staff performance, California Health Alert Network (CAHAN) alerts not being received, Spanish media rumor control, and insufficient information on hourly calls with State agencies (each reported by fewer than 3 respondents).

When prompted for a description of the impact of information sharing and communications challenges, nearly half of these respondents reported that their agency had to divert staff from other tasks to handle communications functions. Other respondents reported that their agency had to make decisions without the most current information available, were unable to send or receive timely information, or had to send or receive the same information multiple times (approximately 20% each). An equal number of respondents indicated there were no consequences as a result of communications challenges.

At the conclusion of the survey, respondents were given the opportunity to relate lessons learned from their experience responding to the tsunami threat. Half of respondents offered comments regarding lessons learned. The topic of communications was cited repeatedly, as “communication with partner agencies” was mentioned in 40% of responses and “communication with the media” and “general communication issues” were each mentioned in 20% of the lessons learned. One respondent noted,

“*We learned that communication with the public and the media is the single most important issue in preparing for any event*.”

“Spanish language communications” was also cited (13%). Other themes included “evacuation” (20%) and “department or emergency operations center issues” (13%).

In addition to lessons learned, respondents were also given the opportunity to share what went well in their organization’s response to the tsunami threat. Half of respondents chose to share such lessons. Of those responses, 60% cited best practices related to “communication with partner agencies”, while “planning” and “alert systems” were each mentioned in 20% of best practices. “Evacuation” was discussed in 13% of responses. One respondent reported that there was “great interaction at the EOC between policy-makers, police, fire, health and emergency personnel”, while another explained that, “many regular updates from our OA [Operational Area] with a NOAA representative were crucial for decision making situational awareness.”

## Discussion

Over the past decade, the importance of public health involvement in emergency response has been increasingly recognized.^8^ However, the roles and responsibilities of public health agencies during emergencies, particularly those without an infectious disease component, are not well defined.^3^ While preparedness planning serves to delineate expected functions prior to an emergency, real events provide an opportunity to observe actual response activities. By studying the experiences of local medical and health governmental agencies during the response to the tsunami threat in California, our research seeks to provide field-based evidence regarding roles and functions of local public health systems. This research focuses on a type of emergency that is rarely experienced in California; however, the findings may also be relevant in preparing for other events where public health agencies play a supporting role in the response effort, as might be common during other natural disasters, severe weather, or mass casualty events.

In response to the tsunami threat in California, we found that emergency management agencies assumed a lead role in the local response efforts. Emergency management representatives reported that their agency was responsible for key activities, including information management, emergency operations coordination, and evacuation activities. While they did not play a lead role, public health and emergency medical services agencies also participated in information management and emergency operations coordination activities. Additionally, these agencies uniquely contributed to public health emergency response functions. Specifically, public health agencies activated surveillance and epidemiology, environmental health, and mental health and psychological support functions. Both public health and emergency medical services agencies also reported participating in mass care and/or the management and distribution of medical materials. If the response to the recent tsunami is any indication, these support activities can be anticipated in planning for future events with similar characteristics to the tsunami threat.

A second research aim was to examine how and when these agencies became aware of the tsunami threat in California. On average, we found that local OES agencies in California became aware of the tsunami threat earlier than their public health and EMS counterparts, and that public health and traditional emergency responders relied on different sources of information for initial notification and maintenance of situational awareness. The average time when respondents were first alerted to the threat through formal channels was approximately 2:30am on Friday March 11^th^, approximately 4.5 hours after the earthquake in Japan. This time to notification poses a challenge to meeting the U.S. Department of Homeland Security’s expectation of developing an initial communications strategy in collaboration with interagency partners within 90 minutes of an incident, as is outlined in the *Target Capabilities List*.^9^ Additionally, nearly one-third of respondents first learned of the tsunami threat through the media, rather than through rapid alerting and notification systems that have seen substantial investment in the last ten years. This indicates that the public may often receive notice of significant emergency events before or at the same time as emergency and public health personnel. Government agencies must continue to develop and maintain the ability to rapidly aggregate and analyze information in order to provide accurate information to a potentially well-informed public in a timely manner.

This fact, coupled with the varying notification times across agency types, suggests that a practice of cross-notification between agencies at the state and local level might be considered as a way to reduce the delay in notification times that, in this instance, some public health agencies experienced. Additionally, because many respondents initially learned about the incident through the media rather than through formal communication channels, governmental response organizations might consider developing the capability to alert and recall their personnel through the TV and radio-based Emergency Alert Systems (EAS) during major events, providing a communication redundancy in case primary alerting and recall methods have failed.

**Limitations.** One limitation of our approach is the use of individual respondents’ notification times as a proxy for agency notification times. It is possible that individual respondents’ notification times may not accurately reflect their agency notification times (i.e. another individual within the agency may have learned about the threat at an earlier time). We attempted to control for this potential bias by recruiting persons in executive positions within their respective organization (e.g., agency administrator or director). Because the functional role of respondents did not systematically vary by agency type, we do not expect that this was an important source of bias.

In the absence of well-defined, universally-accepted performance measures for public health emergency response, we limited our study to characterizing response capabilities in terms of the number and type activated by each agency, and on a qualitative level, asked participants to describe the most significant challenges and lessons-learned. Additional benchmarks for performance would provide greater opportunities for learning and improvement.

Another limitation to our approach is that the survey instrument did not address the concern over radiation exposure in the United States that was raised by radiation leaks at the heavily damaged Fukushima Daiichi Nuclear Power Plant in Japan. There was significant public concern about the potential for radiation exposure in California, an issue that was addressed by communications from California Department of Public Health in conjunction with the California Emergency Management Agency.^10^ While addressing these concerns was not part of the initial emergency response to the tsunami threat, the two events are nevertheless intertwined. Other research on the public health response to radiation concerns in the United States is beginning to emerge.^11^ ^12^ ^13^

## Conclusion

The systematic study of real emergency events has been noted as a gap in the public health preparedness literature. This research characterizes the public health and medical response to the tsunami threat in California following the earthquake in Japan, specifically focusing on two commonly cited areas for improvement: information sharing and defining organizational roles and responsibilities. In response to the tsunami threat in California, we found that emergency management agencies assumed a lead role in the local response efforts. While public health and medical agencies played a supporting role in the response, they uniquely contributed to a number of specific activities. If the response to the recent tsunami is any indication, these support activities can be anticipated in planning for future events with similar characteristics to the tsunami threat. Additionally, we found differences in organizational notification times and sources of notification and situational awareness, suggesting possible areas for future preparedness improvements.

## Endnotes

* Two respondents indicated that they represented two counties and two others noted that their response represented both public health and EMS agency interests for their jurisdiction. These survey responses were counted for each agency and county, as self-reported. As a result, we found one duplicate response. In this case, we chose to include only the response corresponding to the link designated for that organization and operational area.

§ Respondents who could not recall the time at which they first became aware of the threat (n=3) and when they were first formally notified (n=4) were omitted from calculating the average notification time.

? Percentages were calculated among those who reported any communications challenge.

## Competing Interests

The authors declare that they have no competing interests.

## Authors' Contributions

JH, MP and TA conceived of the study and collaborated in the study design. JH and MP coordinated and implemented study recruitment and data collection. JH, MP and TA developed the data collection tools. All authors participated in the data analysis and interpretation, and helped to draft the manuscript. All authors read and approved the final manuscript.

## Appendix A

### Supplemental Material: Survey Instrument

The survey instrument described in this study can be accessed in the following location: http://www.calprepare.com/project4resources.

## References

[1] National Oceanic & Atmospheric Administration (NOAA)/National Geophysical Data Center. Great Tohoku, Japan Earthquake and Tsunami, 11 March 2011 - Runups. Available at: http://www.ngdc.noaa.gov/hazard/honshu_11mar2011.shtml. Accessed February 15, 2012.

[2] National Oceanic & Atmospheric Administration (NOAA). Tsunami Statistics. West Coast and Alaska Tsunami Information. Available at: http://wcatwc.arh.noaa.gov/. Accessed November 21, 2011.

[3] Acosta J, Nelson C, Beckjord E, et al. A National Agenda for Public Health Systems Research on Emergency Preparedness. RAND Corporation; 2009. Available at: http://www.rand.org/pubs/technical_reports/TR660.html.

[4] Nelson C, Lurie N, Wasserman J. Assessing Public Health Emergency Preparedness: Concepts, Tools, and Challenges. Annual Reviews: Public Health. 2007. Available at: http://arjournals.annualreviews.org/doi/abs/10.1146/annurev.publhealth.28.021406.144054. Accessed March 22, 2009. 10.1146/annurev.publhealth.28.021406.144054 17129174

[5] National Biodefense Science Board. Call to Action: Include Scientific Investigations as an Integral Component of Disaster Planning and Response. 2011.

[6] U.S. Centers for Disease Control and Prevention (CDC). Public Health Preparedness Capabilities: National Standards for State and Local Planning. 2011. Available at: http://www.cdc.gov/phpr/capabilities/. Accessed May 9, 2011.

[7] California Department of Public Health and the California Emergency Medical Services Authority. California Health and Medical Emergency Operations Manual. 2011.

[8] Inglesby TV. Progress in Disaster Planning and Preparedness Since 2001. JAMA: The Journal of the American Medical Association. 2011;306(12):1372 –1373. 10.1001/jama.2011.1359 21903817

[9] U.S. Department of Homeland Security (DHS): Target Capabilities List (TCL). 2007.

[10] Backer H, Dayton M. Statement from California’s Department of Public Health and Emergency Management Agency on Risk of Radiation Exposure. 2011. Available at: http://www.cdph.ca.gov/Pages/CDPHCalEMAstatementMarch152011.aspx. Accessed March 5, 2012.

[11] Bobba N. Responding to Public Health Emergencies: Use of Intermediate Management Teams. Presented at: Public Health Preparedness Summit. Anaheim, CA. 2012.

[12] Brinsfield K, Cetron M, McAleenan K, Deitchman S. Returning from Japan: Passenger Radiation Screening at U.S. Airports. 2012.

[13] Schier J, Bronstein A. Surveillance Using the National Poison Data System (NPDS) by the CDC and AAPCC after the 2011 Japan Earthquake and Tsunami. Presented at: Public Health Preparedness Summit. Anaheim, CA. 2012.

